# Gaming behavior and addiction among Hong Kong adolescents

**DOI:** 10.1186/s40405-016-0016-x

**Published:** 2016-07-20

**Authors:** Irene Lai Kuen Wong, Millicent Pui Sze Lam

**Affiliations:** Department of Applied Social Sciences, Hong Kong Polytechnic University, Hung Hom, Kowloon, Hong Kong

**Keywords:** Games, Addiction, Internet, Adolescent students, Prevention

## Abstract

**Objectives:**

Game playing is very popular among Hong Kong teenagers. This study aimed to investigate adolescent gaming behavior and addiction at the Internet cafe, and to explore perceived benefits and harms associated with the activity.

**Methods:**

A convenient sample of 13 male high school students aged 12–15 years (mean age = 13.6 years) were interviewed at two Internet cafes. Young’s (Caught in the net, Wiley, New York, 1998) criteria of Internet addiction were modified to assess gaming addiction.

**Results:**

Internet cafes were described as a safe and ideal rendezvous for gamers. The benefits of gaming included fun and satisfaction, fostering social support and teamwork, meeting new friends and becoming sociable, boosting cognitive techniques and intellectual agility, improved responsiveness and quick thinking. Perceived harms of gaming addiction were reduced time and interest in other important activities, poor academic performance, physical harms and emotional distress, disrupted friendship with non-gaming peers, risked family relationship and financial problems. Five interviewees (38.5 %) could be categorized as pathological gamers and two were problem gamers (15.4 %). The psychological factors associated with gaming addiction include low self-esteem, a strong desire for aggressive and exciting experiences, reliance on gaming to kill time and to obtain satisfaction, coping with problems and negative emotions, and obsession with achieving higher rankings in games. The social and environmental risk factors are accessibility to the Internet cafés, aggressive promotional activities at the Internet cafes, peer pressure, family influence and early gaming experiences, perceived parental approval, lack of parental supervision, and poor family relationship.

**Conclusions:**

The study results throw light on prevention programs.

## Background

The worldwide popularity of gaming, especially among adolescents is shocking. Due to technological advancement, video and online games are increasingly attractive and challenging with beautiful graphics, lifelike images, realistic characters and highly sophisticated game systems. Many youngsters enjoy gaming without experiencing any detrimental consequences. However, some teenagers fail to maintain balance between gaming and school work, family responsibilities and social commitments.

Gaming addiction would negatively impact physical and mental health, cognitive, social, academic and occupational functioning. Pathological gamers exhibit addiction symptoms including salience, tolerance, withdrawal, mood modification, losing control, covering up, and risked significant relationships or opportunities (King et al. 2013; Kuss and Griffiths 2012a; Young 1998). Gaming addiction, which is also widely known as pathological gaming in previous studies, is not included as a psychiatric disorder in the Diagnostic and Statistical Manual of Mental Disorders (fifth edition) (American Psychiatric Association 2013) but it has been listed as a condition for further study.

There were no reports of game addiction until the 1980’s, although the first commercial games had been produced in the early 1970s (Griffiths et al. 2012). Gaming and addiction have become a health concern when home gaming systems (e.g. the PlayStations), online and computer gaming devises were made widely available to numerous customers at affordable prices. A research literature review indicates 1.7 % to over 10 % of the general samples were pathological gamers (Griffiths et al. 2012). Adolescent gaming studies estimate that 2–16 percent of teenager gamers display signs of gaming addiction (Brunborg et al. 2014; Chiu et al. 2004; Gentile 2009; Gentile et al. 2011; Grusser et al. 2007; Jeong and Kim 2011; Ko et al. 2005; Kuss and Griffiths 2012a, b; Rehbein et al. 2010; Wan and Chiou 2007).

Gaming addiction could be a significant health hazard with harmful impact on physical, emotional, mental and social health. It is associated with sleep deprivation, eating irregularities, physical strain and fatigue, obesity, mood disorders, social incompetence and isolation from friends and family (Brunborg et al. 2014: Choo et al. 2010; Young 1998).

Gaming research has documented several individual and environmental factors associated with gaming addiction. More males play video and online games and get hooked than females (Desai et al. 2010; Griffiths et al. 2004, 2012; Gupta and Derevensky 1996; Ko et al. 2005; Rehbein et al. 2010). Approximately 56 % of the gamers are men, and 26 % aged below 18 years (Entertainment Software Association 2015). Male gamers find the activity more exciting and relaxing than their female counterparts, therefore, they are more likely to play excessively (Wood et al. 2004).

Games which involve exercise of power, control and violence attract more males than females (Young 1999). There are numerous aggressive shooter games designed for male adolescent gamers. Young (1999) reported that young men with weak social skills and low self-esteem are most susceptible to gaming addiction because they could design “a powerful persona” within games to win recognition and esteem among players (Griffiths and Hunt 1998; Yee 2006; Young 1999). Many adolescent pathological gamers also have lower academic achievement (Brunborg et al. 2014; Chiu et al. 2004; Skoric et al. 2009) and conduct problems (Brunborg et al. 2014; Rehbein et al. 2010).

Pathological gamers often have poor skills in problem solving and emotion management. Playing games provides excitement, relief and escape from daily stressors and problems (Griffiths 2008; Hussain and Griffiths 2009a; Li et al. 2011; Wood 2008; Wood et al. 2004). Playing games could help modifying moods and negative emotions (Gentile et al. 2011; Griffiths 2008; Hussain and Griffiths 2009b; Wolfling et al. 2008; Wood 2008; Wood et al. 2004), consequently the players are likely to indulge in gaming to excess (Jacobs 1986).

Cognitive factors such as distorted perception of one’s intelligence and gaming skills may contribute to pathological gaming (King and Delfabbro 2014). Many pathological gamers have positive appraisal of their intelligence and gaming skills but a negative view of their social competence in interpersonal interactions (Gentile et al. 2011; Zhong 2011). They may also have a strong desire to seek new sensations and experiences (Mehroof and Griffiths 2010).

Adolescents are susceptible to peer influence (Beard 2005). Modeling may play a role in pathological gaming. Teenagers watch and imitate peers around them playing games to seek relaxation, to meet others, and to cope with upsetting emotions and problems. They are also compelled to meet peer expectations and pressure to continue playing hours after hours to keep up with the game, especially in the Massively-Multiplayer Online Role-Playing Games (MMORPGs). The MMORPGs could be a risk factor of gaming addiction as there seems to be no end to these games. The gamers are emotionally attached to the gaming group, and feel obliged to keep playing (Hussain and Griffiths 2009a; Yee 2006; Zhong 2011).

The environment where gaming activities take place would increase the potential for developing problems. Besides the comfortable home, some youngsters prefer playing games at the Internet café where their peers hang around (Beard 2005). Extended gaming at the Internet cafes is likely when these teens have to keep playing under peer pressure.

There is a paucity of research on adolescent gaming in Hong Kong. Much of the currently available data are derived from studies on problematic Internet use. Research results reveal that 4.5–16 % of adolescent compulsive Internet users were mainly involved in playing games, electronic communication and social networking (Breakthrough Limited 2003; Chak and Leung 2004; Fu et al. 2010; Hong Kong Christian Service 2009). Wang et al. (2014) reported 94 % of 503 high school students surveyed had played video or Internet games, and 15.6 % had a gaming addiction. This is the only recent local study focusing on youth gaming but no clarification of gaming venue has been made.

We argue that gaming at the Internet café could be potentially more hazardous than home gaming. Driven by the profit making motive, the café managers would not stop young excessive gamers to play, and venue-based safeguards against gaming addiction do not exist. It is more feasible to implement measures to discourage or stop youth gaming at home (e.g. removing gaming devices, suspension of the Internet service, providing parental supervision and familial intervention, etc.).

This exploratory study helps filling the current research gap by conducting interviews with male teenagers who played games at the Internet café. To our knowledge, this is the first qualitative study on adolescent Internet café gaming from the participants’ perspective. The study results would shed light on prevention of adolescent gaming addiction. The aim of the study is threefold: (a) to examine the reasons for playing games at the Internet cafe when home gaming is an option available to many Hong Kong adolescent students, (b) to investigate the teenagers’ perceptions of benefits and harms associated with gaming at the Internet cafes, and (c) to identify psychosocial risk factors of gaming addiction.

## Method

### Procedure

A convenient sample of 13 male gamers was recruited from two Internet cafés. Participation was anonymous and voluntary. A total of 23 male junior graders were approached and invited to participate in the study but only thirteen agreed to be interviewed. The response rate was 56.5 %. The major refusal reasons were being too busy with gaming or being unable to squeeze time for an interview due to other obligations.

After clarification of research purposes and obtaining written consent from the participants, semi-structured interviews were conducted either inside the Internet café with the manager’s approval or near the entrance when the gamers were leaving. On average, the interviews lasted for 30 min. A trained and experienced researcher used a Chinese interview guide to collect data. Extensive notes were taken during the interviews while two interviews were taped with the interviewees’ permission.

The merit of semi-structured interviews is the feasibility to collect data via focused, conversational, two-way communication using pre-determined questions while allowing the participants to speak freely beyond the interview guide (Cohen 2006; Laforest 2009).

### Participants

All the interviewees were male junior graders aged between 12 and 15 years (mean age = 13.6 years; SD = 1.2). Six (46.2 %) were 14 years old, three (23.1 %) were thirteen, two were fifteen (15.4 %), and another two were twelve (15.4 %). Six students were in grade seven (46.2 %) and eight (46.2 %) respectively and one (7.7 %) was in grade nine.

### Instruments

The Chinese interview guide comprises four major sections:

1. A demographic section gathering data on the participant’s age and school grade;
2. The interviewee’s reasons for playing games at the Internet cafe and his gaming habit, including the preferred games (e.g. violent games, role-playing games, etc.), gaming frequency and time spent playing games in each visit to the Internet cafe;
3. Young’s (1998) Internet Addiction criteria were modified to assess gaming addiction. These criteria are gaming preoccupation, tolerance and withdrawal symptoms, loss of control, lying, escape from problems or seeking mood modification, extended gaming, and jeopardized significant relationships or educational opportunity. As recommended by Young (1998), endorsement of five or more of the criteria suggests pathological gaming, identification of three or four criteria indicates problem gaming. Low-risk gamers would endorse one or two criteria, and recreational gamers would not endorse any criteria. In this study, to enhance comparison between gamers who were addicted to game playing and those who were not hooked, the recreational and low-risk gamers are combined to become the “non-problematic” gamers. The pathological and problem gamers are combined to become the “problematic” gamers.
4. The interviewee’s perceptions of benefits and negative consequences of gaming;
5. Questions designed to collect information for investigating factors associated with gaming addiction. Interviewees were asked to present self-reports on academic performance, coping with problems and negative emotions, perceived parental support, and relationship with family and peers.

### Data analysis

**Fig. 1:**
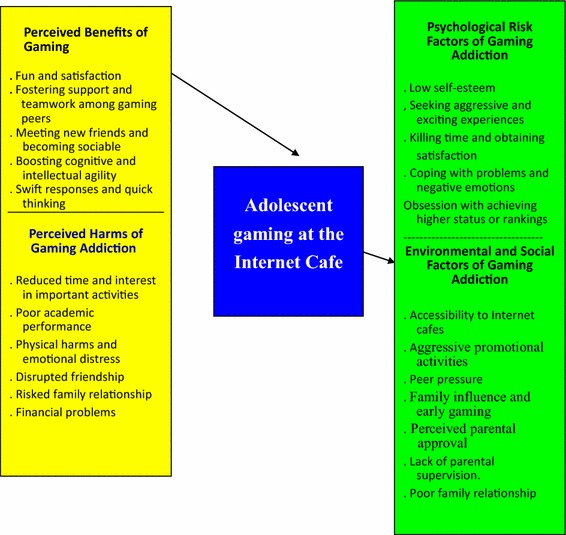
Main and sub-themes emerged from the study

The interviews generated information which was first transcribed and then analyzed by thematic analysis (Patton 2002). After examination of the transcribed text, the researchers identified both main and sub-themes emerged from the study (Fig. 1). We also used quotations from the original text to illustrate the sub-themes.

## Results

### Early gaming experiences

All the interviewees began playing video and online games at the Internet cafe while attending primary school before twelve (mean age = 9 years). Five started gaming between 10 and 11 years, while eight between 7 and 9 years. Eight were coached by their friends to play the first game, and five were encouraged to play by their classmates. Seven (53.8 %) played games at the Internet cafés at least once a week, three (23.1 %) did so only on weekend and during the public holidays, two (15.4 %) played on a weekday basis, and one (7.7 %) played only once a month. They spent at least 1 h on gaming in each visit to the Internet cafe.

### Home gaming

Nine teenagers (69.2 %) also played games every day at home, three played one to five times a week, only one interviewee did not play any games at home because Internet service was not available.

### Reasons for gaming at the Internet cafes

All agreed that the Internet cafes were “safe, comfortable” and “ideal rendezvous” for gamers. They were often tempted by the “new and challenging games” which were only available at the Internet cafes. Many also enjoyed the “snacks and drinks” provided at “attractive prices”. Several even ran to play and eat at the nearest Internet cafe with their gaming peers during school lunch break.

### Perceived benefits of gaming at the Internet café

Many interviewees reported the following benefits of playing games at the Internet café: fun and satisfaction, fostering support and teamwork among gaming peers, meeting new friends and becoming sociable, boosting cognitive techniques and intellectual agility, improved responsiveness and quick thinking,

### Fun and satisfaction

All were introduced to gaming at the Internet cafe by their friends or classmates. With the exception of one interviewee, all played games at home as well. All agreed that, “Games are simply good fun providing great enjoyment.” They loved the excitement and satisfaction derived from playing and winning games. Many said, “No other activities could be more interesting”.

### Fostering support and teamwork among gaming peers

All interviewees preferred playing games with their gaming peers than playing alone. Involvement in the multiplayer games at the Internet cafes became a peer group activity which would foster support and teamwork. They explained, “We form strategic alliance to fight against the monsters and enemies”. A teen concurred that “my team would rescue me whenever there is any danger of being attacked by our opponents. “I love cooperative games in which support, teamwork and skills are the keys to winning and victory.” Many interviewees agreed that “multiplayer and cooperative games help strengthening support, team spirit and peer group identity” which were of premier importance during the developmental stage of adolescence.

### Meeting new friends and becoming sociable

Many cooperative games could be very sociable because they involve playing games with other people including strangers. Several players treasured the opportunity to meet new friends at the Internet cafes. They said, “Gaming helped breaking the ice with strangers met at the Internet cafe and creating topics for sharing and discussion.” A gaming team of three noted that “We share our recent gaming experiences, and discuss strategies to win weapons and defeat the enemies.” They also established social relationships through online interactions. A boy said, “I met teenager gamers from other countries. It is easy to make new friends living outside Hong Kong.”

### Boosting cognitive techniques and intellectual agility

Many games target memory, reasoning and logic. These games were valued as cognitive boosters by the interviewees. Seven respondents reported having experienced improved memory accompanied by better reasoning and intelligence as a result of gaming. Players of intellectual games noted, “We become more clever as we are frequently tested on how to work out strategies to beat other gamers.” One adolescent explained that, “the activity also helps the players to develop creativity and use imagination to create roles and characters in the games.”

### Improved responsiveness and quick thinking

Many remarked that, “In the tense ever-changing and extraordinarily competitive environment of the simulated world, one learns to think quickly and respond swiftly in order to beat the monsters or opponents, and to get the weapons, treasures and awards.” All noticed how fast action games helped improving eye-hand coordination and intelligence.

### Perceived harmful consequences of gaming addiction

Perceived deleterious effects of gaming addiction were reduced time and interest in other important activities, poor academic performance, physical harms and emotional distress, disrupted friendship with non-gaming peers, risked family relationship and financial problems.

### Reduced time and interest in other important activities

Majority admitted they had spent more time on playing games than study and other important activities. Many reported, “Only games seem to attract me, hence, I have been investing increasing amount of money and time on playing games”. All noted that “gaming could take a lot of hours, and that time could be spared for sports, family activities or doing something else with friends.”

### Poor academic performance

Almost all the interviewees agreed that “games are much more interesting and important than completing school assignments.” Six admitted they had keen interest in playing games but disliked doing school assignments and revision. The gaming habit interfered with time management producing negative impact on academic results. One interviewee said, “I am weak in time management. I often play for hours and do not have time to finish school work.” Another interviewee noted that “I do not like my study which is so boring and difficult, and I would turn to playing games”. The problematic gamers were doing badly in school assignments, tests and examinations.

### Physical harms and emotional distress

Playing games for extended periods of time without taking a break was very common among the interviewees. Many admitted, “We feel exhausted but it is costly to take a break. We have to concentrate and respond to the tense and competitive environment of the simulated world.” As a result of repetitive actions, staring and sitting still in front of the gaming machine for hours, majority experienced dry eyes, blurred vision, dizziness, muscular stiffness in the shoulders, finger and waist strain.

The pathological gamers reported more severe physical damages including headache, severe back pain, stomach ache due to skipping meals for gaming, sleep disturbances, and feeling anxious or depressed due to gaming-related problems.

Majority of the interviewees also experienced emotional ups and downs due to gaming. Winning games would bring a sense of achievement and satisfaction but losing would produce negative affect such as feeling upset, angry, disappointed and nervous. Several interviewees said, “We would throw temper after losing in games to ventilate ill feelings.” They also recognized they often became irritable and impulsive after playing aggressive video and online games.

### Disrupted friendship with non-gaming peers

There are games designed for single players. Interviewers who played alone for hours were often criticized for isolating themselves from their friends, especially the non-gaming peers. They knew they maintained less contact with friends who did not love playing games but they disagreed that they would lose them one day due to gaming.

### Disrupted family relationship

All interviewees claimed that their parents were aware of their game playing habit at the Internet cafe. Eight reported, “My parents don’t like me playing games there.” Six confirmed, “We spend less time with our family members in order to save time for playing games with friends.” Five recognized that their relationship with their family members got worse due to gaming. Several were angry with their parents who had taken offensive measures to control or interfere with their gaming behavior. A teen said, “I go out to play games without telling my parents. I know they would stop me from going to the Internet cafe.” Many hated their parents’ nagging and endless complaints about their game playing habit. They realized parent–child communication and relationship worsened due to conflicts over the activity. An interviewee said, “I quarrel with my parents about games almost everyday….they hit me and drive me away when they go mad. I fight back!” Another youngster reported, “My family relationship is very poor but I don’t care…I shall go on playing games anyway.”

### Financial problems

Playing games at the Internet café could be costly. Interviewees spent a considerable proportion of their pocket money on the activity. Playing games for unanticipated long hours would incur great expenses interfering with the original budget. Majority admitted that they had spent too much money on gaming causing difficulty in meeting competing financial needs (e.g. eating lunch, traveling to and from school by public transport, and joining extra-curricula activities). A boy said, “I don’t have money to buy new books due to overspending on games.” Some would resort to using the red packet money given by adults at the Chinese New Year to cover the essential school expenses.

To conclude, the interviewees clearly reported the benefits and the detrimental impacts associated with the gaming activity. When they were asked if they would cut down or quit gaming to reduce gaming-related harms, none were willing to cut down or give up the activity. Many interviewees said, “Some teens are addicted to gaming but I don’t play as excessively as they do. The chance for me to get hooked is extremely slim.” They emphasized their ability to control gaming rather well, and could stop playing games anytime they wished. Their awareness of risk was low but perceived control over gaming appeared to be high.

### Gaming addiction

Young’s (1998) criteria of Internet addiction were adapted to assess gaming addiction. Five interviewees (38.5 %) could be categorized as pathological gamers who met five or more gaming addiction criteria. Two could be classified as problem gamers who endorsed three (7.7 %) and four criteria (7.7 %), respectively. Two teens met two criteria (15.4 %) and four endorsed only one criterion (30.8 %). These six adolescents who endorsed only one or two criteria were not addicted to games. They were grouped together to become the “non-problematic gamers” to enhance comparison with the “problematic gamers” who met at least three criteria.

Table 1 summarizes the symptoms of gaming disorder reported by the interviewees. The four most commonly exhibited addiction symptoms are: (a) having jeopardized or risked significant relationships because of gaming at the Internet cafe (84.6 %), (b) feeling preoccupied with games (53.8 %), (c) playing games with increasing amounts of time to achieve satisfaction (53.8 %), and (d) playing games for longer time than originally planned (53.8 %).

**Table 1:** Endorsement of gaming addiction criteria

Criteria of gaming addiction	N (%)
1. Feeling preoccupied with playing games at the Internet café	7 (53.8 %)
2. Playing games at the Internet café with increasing amounts of time to achieve satisfaction	7 (53.8 %)
3. Repeatedly made unsuccessful efforts to control, cut back, or stop gaming at the Internet café	3 (23.1 %)
4. Feeling restless, moody, depressed, or irritable when attempting to cut down or stop gaming at the Internet café	4 (30.8 %)
5. Playing games at the Internet café longer than originally planned	7 (53.8 %)
6 Having jeopardized or risked significant relationships, educational opportunity because of gaming at the Internet café	11 (84.6 %)
7. Having lied to family members, therapists, or others to conceal the extent of gaming involvement	4 (30.8 %)
8. Playing games at the Internet café to escape from problems or to relieve dysphoric mood	2 (15.4 %)

Almost one third (30.8 %) felt restless, moody, depressed, or irritable when attempting to cut down or stop gaming at the Internet cafe. Four (30.8 %) lied to family members or others to conceal the extent of gaming involvement.

### Psychological risk factors of gaming addiction

Several psychological factors associated with pathological and problem gaming have been identified. These include low self-esteem, a strong desire for aggressive and exciting experiences, reliance on gaming to kill time and to obtain satisfaction, coping with problems and negative emotions, and obsession with achieving higher status or rankings in games.

### Low self-esteem

Gamers with low self-esteem are more vulnerable for being hooked to game addiction (Li et al. 2011). The pathological and problem gamers agreed that they had low self-worth and described themselves as school failures with poor academic results. Feeling good at nothing in study, gaming helped boosted self-worth. A gamer said, “I feel helpless and useless when talking about school and study….I feel less worthless when my peers tell me I am a useful member of the gaming team. They praise me for my gaming skills.” The gamers’ self-esteem and image improved with peer support, and when they boasted about their gaming skills and knowledge on video and online games. They believed they were clever at playing games which tested intellectual competence, quick response and motor skill.

### A strong desire for aggressive and exciting experiences

The problematic gamers loved aggressive video and online games which would allow them to shoot and kill freely. The virtual nature of the games let them perform extremely violent and aggressive actions such as fighting, shooting and killing with support and appreciation from their gaming friends. A gamer said, “Killing is not allowed in real life but I could kill and gun down as many as I wish in the games.” Many agreed they “enjoyed aggressive behavior like battering, shooting and killing in games.” They also admitted having a strong desire for seeking excitement and sensation from playing violent and aggressive games.

### Reliance on gaming to kill time and obtain satisfaction

The gaming addicts reported that gaming was the only favorite pastime. They were puzzled what to do and how to kill time and boredom without video and online games. They admitted they might have relied too much on gaming to obtain a sense of satisfaction in daily life. They treasured the rarely found success and achievement through accomplishment of gaming tasks, gaining bonus or game points, seizing treasures, having status or level promotion. Such successful experiences were hard to find in everyday life. A boy said, “I feel proud, very satisfied and successful when I win in challenging games”. The sense of achievement and satisfaction fueled their interest and passion to continue playing games at the Internet cafe.

### Coping with problems and negative emotions

The problematic gamers were incompetent in regulating negative emotions and coping with stress and problems (Griffiths 2008; Wood 2008; Wood et al. 2004). Several claimed that “I don’t know how to cope with anger and frustration. I play games or go to sleep when there are troubles and problems.” They were leading an unhappy life. They resorted to gaming to relieve negative affect of anger, frustration, anxiety and depression. A youngster said, “When I am angry or upset, I will turn to playing games with my friends…. I often feel much relieved after gaming.” Another claimed that “when I win a game, I feel very happy and successful….all my blues and troubles have gone.” When they concentrated in the games, they would forget sorrow and problems.

### Obsession with achieving higher status or rankings in games

The addicted gamers felt that they were becoming smarter and more intelligent due to gaming. They spent extended period of time in playing games in order to achieve higher status and rankings. Such obsession with level or status promotion has been rationalized by the players. Many knew that they were obsessed with level promotion but they seemed to enjoy having such an obsession as level promotion made one feel better of oneself, especially when the gaming friends were around. A gamer claimed that “adjustment to a lower level could be tuff and humiliating in the group games because this may imply failure and losing face.”

### Social and environmental risk factors of video gaming addiction

The social and environmental factors associated with gaming addiction are accessibility to the Internet cafes, aggressive promotional activities at the Internet cafes, peer pressure to continue playing, family influence and early gaming, perceived parental approval, lack of parental supervision, and poor family relationship.

### Accessibility to the Internet cafes

When the study was conducted, there were many Internet cafes in the densely populated regions in Hong Kong. All the interviewees agreed that “accessibility to the Internet cafes might have increased their interest in games”. The problematic gamers said, “We run to the nearest Internet café to play games during the short school lunch break because it is close to the school.” Many Internet cafes provided highly affordable fast food and drinks during lunch time to attract young people and students. A lunch time regular gamer explained, “Our desire for gaming and eating could be satisfied simultaneously at a very attractive fee.” Many would also spend at least a couple of hours on games after school and during weekends and public holidays because the Internet cafes were in the neighborhood.

### Aggressive promotional activities at the Internet cafes

In order to attract adolescent and adult customers, many Internet cafes provide attractive food and game special offers and gaming coupons at lower prices. Other aggressive promotional activities were also adopted, including lower hourly rates for extended time of playing, colorful posters promoting new games and prizes for the winners of gaming competition, etc. Several problematic gamers were aware of how these promotion and marketing strategies had increased their gaming motivation, gaming frequency and the duration of playing games in each visit to the Internet cafe. They said, “We take advantage of these special offers to play more frequently and stay longer time on the games until getting close to bankruptcy or exhaustion. Yes, quite obsessed but still good fun.” When the special offers ended, they were forced to cut down gaming reluctantly because of money problems, and they reported withdrawal symptoms of feeling restless, depressed and irritable.

### Peer pressure to continue playing games

Group games and the massively multiplayer role playing games have been identified as risk factors of addiction (Hsu et al. 2009; van Rooij et al. 2011) because the gamers are reluctant to stop playing under peer pressure. A teenager explained that “we always form alliance to fight against the opposing team and enemies. I am forced to go on even after playing for a very long period of time. I don’t want to let my team members down. They will be angry and will scold me if I quit.” Another interviewee shared similar experience of playing group games overnight. He admitted that “it was really excessive and exhaustive indeed, but as my gaming friends did not want to leave, I could not go either.” Several concluded that they were compelled to continue playing even when they were losing in the games for fear of being blamed.

### Family influence and early gaming

Ten interviewees reported having family members who loved playing games. Six claimed that their siblings were fond of gaming, and four helped their parents to download games. A boy said, “My mother plays Tetris. She asks me to download and install gaming applications to her iPad”. Another interviewee said, “Both my mother and my sister like playing games installed in their smart-phones”. Another teenager’s mother liked playing games which could be found from the QQ. The problematic gamers were exposed to a gaming family environment where parents and siblings were also fond of playing games either online or offline. Playing video and online games either alone or with family members became an acceptable activity in early family life. All the problematic gamers began gaming before the age of 9 years.

### Perceived parental approval

All the interviewees reported having obtained parental approval of playing games at the Internet cafes. Most gamers convinced their parents that the Internet cafes were safe and comfortable playing and learning venues where cognitive and intellectual ability could be trained. A boy said, “My mother encouraged me to play games that would improve intellectual ability.” Children tried to convince their parents that gaming at a safe Internet cafe was similar to joining an after school learning program where they could play, learn and eat at affordable prices. A player remarked that “My parents did come here to have a look to ensure it is safe. They allow me to play games for hours on weekend….if they forbid me playing games here, I would leave much earlier to avoid being criticized.” Perceived parental approval seems to reinforce extended gaming.

### Lack of parental supervision

Many parents had to work long hours to make a living, and could hardly provide effective supervision on their children’s gaming frequency, gaming time and expenditure. One youth noted that “my parents work very late in the evening. They don’t know where I usually go and what I do after school. Gaming at the Internet café is good fun. I hate staying home to be alone and be bored to death.” Another boy reported, “My mum emphasizes that it is okay to play occasionally at the Internet cafes, as long as I don’t go there frequently.” These parents could barely monitor their children’s gaming behavior. A teenager said, “My mother reminds me again and again of having self-control over gaming. She was silent upon learning that I do frequently play video and online games at a nearby Internet café.” Several admitted that they often spent much more time on playing games than originally intended to when parental control on gaming is minimal due to work commitments. Interviewees who lacked parental supervision were prone to playing games excessively. Those who had parental guidance were more likely to stop playing games according to the agreed time, and ran home for dinner and other familial obligations.

### Poor family relationship

The problematic gamers felt being neglected by their parents and family members who failed to provide them with emotional support and help. A youngster said, “My parents and grandma only love my younger brother. No one loves me and helps me in this family.” Another felt annoyed with his parents who yelled at him over unfinished school assignments and games. They quarreled everyday. A pathological gamer tried to cope with unhappy family life by increasing gaming time and frequency to forget family troubles. A vicious circle developed with an increasing desire to play games when family distress and conflicts were escalating, and obsessive gaming behavior would cause further damages to family relationships. Another pathological gamer noted, “My family has given me up after years of battling over gaming and poor school results.…, I don’t want to stay in this family. I feel better only when I am playing games with my friends at the Internet cafe. We don’t like our family. When we concentrate in playing games, all the troubles will vanish.”

## Discussion

This qualitative study increases our understanding of adolescent gaming behavior and addiction at the Internet café. However, there are several limitations in this study, including using a small convenience sample of 13 male teenagers, and the unverified reliability of the interviewees’ self-reports. Hence, the study results may not be transferred to female gamers and players who do not visit Internet cafes for gaming purposes. Future research should recruit larger samples, and include female gamers for comparison and discussion. If research funds and resources are sufficient, longitudinal studies are most suitable for investigating the onset, development, maintenance and relapse of this behavioral addiction.

To our knowledge, this study is the first qualitative investigation of youth gaming behavior at the Internet café. Several new findings are found, including: (a) perceived benefits and harms associated with gaming, (b) new risk factors of gaming addiction (i.e., perceived parental approval, and aggressive promotional activities organized by the Internet café managers), (c) and a higher gaming addiction estimate (38.5 %) found in a commercial gaming environment where safeguards against addiction did not exist.

Previous studies report a lower rate of gaming addiction (e.g. 15.6 % noted in a survey conducted by Wang et al. 2014). We argue that home gaming might be relatively safer than Internet café gaming because parental supervision and familial intervention (e.g. parental control of gaming duration and frequency, removing gaming devises, and suspension of Internet services, etc.) seem to be more available than help to be provided by the Internet café operators. This exploratory study provides support for conducting future research to verify if the prevalence rate of gaming addiction is higher at the Internet café than at home.

Consistent with early studies, most of the interviewees experienced physical discomforts and health problems (e.g. dry eyes, blurred vision, dizziness, finger and waist strain, back pain, and feeling exhausted, anxious, irritable or depressed) because of extended period of gaming (Brunborg et al. 2014; Choo et al. 2010; Desai et al. 2010). Many gamers also neglected other important activities, argued with their parents over games, and reported unsatisfactory school results. Several even had financial problems due to over spending on playing games at the Internet cafe. None were willing to change their gaming habit. They were confident of having control over the activity. It seems that perceived control over gaming was high but awareness of risk associated with the activity was low. Tertiary prevention programs for pathological and problem gamers may include changing illusions of control over gaming, treatment of gaming addiction symptoms, physical damages and emotional distress. It is also necessary to design primary preventive efforts which promote awareness of risk and harms associated with the activity.

Replicating past research, the pathological and problem gamers were more likely to use games to alleviate negative emotions (e.g. Gentile et al. 2011; Hussain and Griffiths 2009b) and to cope with real life problems (Griffiths 2008; Hussain and Griffiths 2009a; Li et al. 2011; Wood 2008; Wood et al. 2004). They reported poor academic results, low self-esteem, disrupted family relationship and lack of parental supervision. Playing games with peers at the Internet cafe might have met unfulfilled but important psychosocial needs for companionship, social support, and creating experiences of achievement and satisfaction. It is necessary to help these young people to find other health-enhancing activities to meet these essential human needs. Secondary preventive programs should be designed and target specifically these young gamers who seem to be more vulnerable to gaming addiction.

This study provides evidence for verifying that the Internet cafe and the multiplayer group games (e.g. the MMORPGs) could be the risk factors for developing gaming addiction (Hsu et al. 2009; van Rooij et al. 2011). The problematic gamers had developed an emotional attachment to their gaming friends who frequented at the Internet cafes. They allied to play cooperative games with a strong sense of loyalty to the other team members. They felt obliged to keep playing even to excess. The promotional activities at the Internet cafes also encouraged gamers to play for long hours. Responsible gaming measures should be implemented at the Internet cafes with warning messages on gaming addiction. Information on professional help should also be provided.

One study result that warrants special attention is pathological and problem gamers were in lack of parental supervision on their gaming habit. It is necessary to develop parent awareness programs and to help parents to monitor children’s use of Internet services and gaming systems within and outside the home environment.

## Conclusions

This study indicates problematic gaming is the result of a complex interplay between the activity itself, the gaming venue, familial and psychosocial factors (Griffiths and Wood 2004). From a public health perspective, it is important to provide early education to increase children’s awareness of risk, harms and signs of gaming addiction as all the interviewees in this study began playing games at an early age during primary school years (mean age = 9 years). More research is needed to increase our understanding of children and adolescent gaming behavior, addiction and resiliency.
